# Differential Regulation of Pre-Harvest Sprouting by *OsERF1* and *OsERF94* Through Hormone Signaling and Metabolic Reprogramming in Rice

**DOI:** 10.3390/ijms27135915

**Published:** 2026-06-30

**Authors:** Yu-Jin Jung, Jong-Hee Kim, Jin-Young Kim, Jiyun Go, Hak-Soo Kim, Sang-Mun Jung, Kwon Kyoo Kang

**Affiliations:** 1 Division of Horticultural Biotechnology, Hankyong National University, Anseong 17579, Republic of Korea; yuyu1216@hknu.ac.kr (Y.-J.J.); jonghee014@hknu.ac.kr (J.-H.K.); zino@hknu.ac.kr (J.-Y.K.); haksoolove@naver.com (H.-S.K.); mocgidan@hanmail.net (S.-M.J.); 2 Institute of Genetic Engineering, Hankyong National University, Anseong 17579, Republic of Korea; 3 Department of Bio-Environmental Chemistry, College of Agriculture and Life Sciences, Chungnam National University, Daejeon 34134, Republic of Korea; jy.go2369@gmail.com

**Keywords:** PHS, rice, *OsERF1*, *OsERF94*, ABA signaling, carbohydrate metabolism

## Abstract

Pre-harvest sprouting (PHS), the premature germination of grains on the mother plant, causes substantial yield loss and grain-quality deterioration in rice under humid conditions. Although seed dormancy and germination are largely controlled by hormonal balance, the transcriptional mechanisms linking hormone signaling with metabolic adaptation during PHS remain unclear. In this study, we investigated the roles of two ethylene-responsive factor transcription factors, *OsERF1* and *OsERF94*, in rice PHS regulation using CRISPR/Cas9-mediated knockout lines, together with physiological, gene-expression, and metabolite analyses. The *oserf1*-KO mutant showed reduced seed dormancy and increased germination under PHS-inducing conditions, accompanied by altered expression of abscisic acid- and gibberellin-related genes. In contrast, the *oserf94*-KO mutant exhibited enhanced dormancy and reduced germination, with decreased expression of hypoxia-responsive fermentation genes and impaired carbohydrate mobilization, as indicated by reduced soluble sugar and ethanol accumulation and increased starch content. These results suggest that *OsERF1* contributes primarily to hormone-mediated dormancy maintenance, whereas *OsERF94* supports metabolic activation required for germination under high-moisture conditions. Collectively, this study proposes a dual regulatory framework in which hormonal control and hypoxia-associated carbon metabolism coordinately determine rice PHS susceptibility.

## 1. Introduction

Rice (*Oryza sativa* L.) is one of the most important cereal crops worldwide, and its stable production is essential for food security under increasingly unstable climatic conditions. During grain maturation and harvest, prolonged rainfall and high humidity can induce PHS, which is defined as the premature germination of grains while they remain attached to the mother plant [1]. In rice, PHS reduces seed viability, deteriorates grain quality, and causes substantial economic losses, particularly in humid cultivation regions [1]. PHS susceptibility is closely associated with seed dormancy, an adaptive trait that prevents mature seeds from germinating under unfavorable environmental conditions [2,3]. Genetic studies have identified several dormancy- and PHS-associated loci in rice, among which *Seed dormancy 4* (*Sdr4*) has been characterized as a major regulator of seed dormancy [4]. Subsequent population-level analysis further showed that *Sdr4* strongly contributes to PHS resistance and adaptation to local climatic conditions in Asian cultivated rice [5]. Recent advances have further expanded this genetic framework through fine-mapping of PHS-tolerance loci, CRISPR/Cas9 editing of *OsABA8ox*, and functional characterization of *OsAAH* in rice, while recent reviews have synthesized emerging PHS and hormone-regulatory mechanisms across crop species and rice [6,7,8,9,10]. At the hormonal level, the transition from dormancy to germination is largely regulated by the antagonistic actions of abscisic acid (ABA) and gibberellins (GAs), with ABA promoting dormancy maintenance and GA promoting dormancy release and germination [11,12]. Comparative analyses of dormant and non-dormant rice cultivars have also shown that dormancy status is associated with distinct expression patterns of ABA and GA metabolism genes and corresponding changes in hormone levels during seed development and imbibition [13]. In addition, ethylene can modulate seed germination through interaction with ABA signaling, suggesting that PHS-related germination is governed by hormonal crosstalk rather than by ABA–GA balance alone [14,15]. However, the transcriptional regulators that connect ethylene-associated signaling with ABA-related dormancy control during rice PHS remain insufficiently understood. Successful germination under PHS-inducing environments also requires metabolic competence. High humidity or submergence-like conditions may restrict oxygen availability within seed tissues, thereby limiting mitochondrial respiration and requiring metabolic adjustment [16,17]. Under oxygen-deficient conditions, plants activate glycolysis and anaerobic fermentation to sustain ATP production and maintain cellular redox balance [16,17,18]. Rice is notable among cereals for its ability to germinate under low-oxygen or even anaerobic conditions, and this capacity depends on the mobilization of stored carbohydrates into soluble sugars that can support fermentative metabolism during early growth [19]. Therefore, PHS should not be viewed only as a failure of dormancy maintenance; it also requires carbon mobilization and energy metabolism sufficient to support germination under humid and potentially hypoxic conditions. Ethylene-responsive factor (ERF) transcription factors, members of the APETALA2/ethylene-responsive factor (AP2/ERF) superfamily, are important regulators of hormone signaling, environmental stress responses, and developmental processes [20,21]. In rice, *SUB1A*, an ethylene-response-factor-like gene, confers submergence tolerance, demonstrating that ERF-mediated regulation can play a major role in low-oxygen adaptation [22]. In addition, group VII ERFs have been implicated in plant oxygen sensing and hypoxia-responsive transcriptional networks [23,24]. These findings suggest that ERF transcription factors are plausible candidates for linking ethylene-associated signaling, environmental moisture stress, hormonal regulation, and hypoxia-related metabolic responses. Nevertheless, the specific roles of individual rice ERF genes in PHS regulation remain poorly characterized, particularly in relation to their possible association with hormone-mediated dormancy maintenance and hypoxia-associated metabolic activation.

In this study, we investigated the roles of two rice ERF loci, Os04g0546800 (transcript Os04t0546800-01; protein accession XP_015636763.1), designated in this study as OsERF1, and Os04g0547600 (transcript Os04t0547600-01; protein accession XP_015635116.1), designated in this study as OsERF94, in the regulation of pre-harvest sprouting. Using CRISPR/Cas9-mediated knockout lines, we examined PHS-associated sprouting phenotypes, hormone- and hypoxia-related gene expression, and changes in carbohydrate metabolism. We hypothesized that *OsERF1* and *OsERF94* contribute to PHS through distinct regulatory processes associated with dormancy maintenance and metabolic activation, respectively. Our findings provide a framework for understanding how ERF-associated hormonal and metabolic pathways influence PHS in rice.

## 2. Results

### 2.1. Structural Conservation of OsERF1 and OsERF94 Supports Potential Functional Divergence

**Figure 1:**
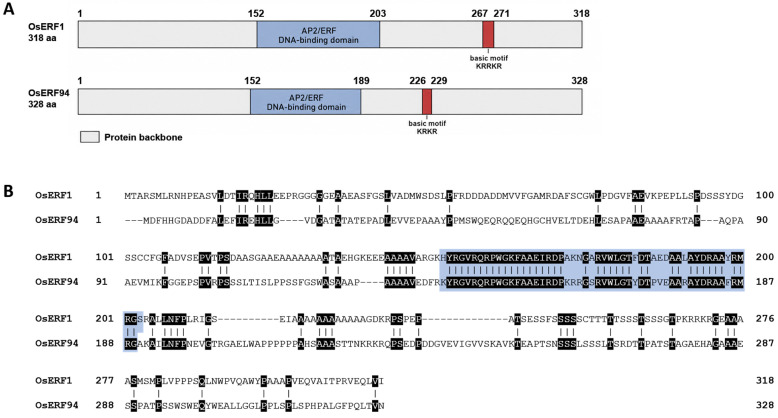
Structural and sequence conservation analysis of OsERF1 and OsERF94. (**A**) Schematic representation of the predicted protein structures of OsERF1 and OsERF94. The AP2/ERF DNA-binding domain is shown in blue, and the putative basic motif is indicated in red. Amino acid positions are indicated above each protein model. (**B**) Full-length amino acid sequence alignment of OsERF1 and OsERF94. Conserved residues are highlighted by shaded boxes, with darker shading indicating higher sequence conservation. The AP2/ERF DNA-binding domain is highlighted in blue.

*OsERF1* (Os04g0546800) and *OsERF94* (Os04g0547600) encode ERF-type transcription factors consisting of 318 and 328 amino acids, respectively (Figure 1A). Protein architecture analysis showed that both proteins contain a single AP2/ERF DNA-binding domain in the central region, together with a short basic motif located outside the AP2/ERF domain (Figure 1A). The basic motif was identified as KRRKR in OsERF1 and KRKR in OsERF94, suggesting that both proteins possess conserved basic amino acid-rich regions. Pairwise sequence alignment of OsERF1 and OsERF94 showed that sequence conservation was mainly concentrated within the AP2/ERF DNA-binding domain, whereas the N- and C-terminal regions were more divergent (Figure 1B). Within the AP2/ERF domain, both proteins retained the conserved YRG and RAYD elements characteristic of AP2/ERF-family DNA-binding domains. The YRG-containing region contributes to DNA recognition, whereas the RAYD-containing region forms part of the conserved structural framework of the domain, including its α-helical component [25,26]. In contrast, sequence similarity outside the AP2/ERF domain was relatively low, with multiple gaps and variable amino acid regions observed between the two proteins. To further evaluate domain conservation, the full-length sequences of OsERF1, OsERF94, and representative ERF family proteins from other plant species were aligned (Supplementary Figure S1). The AP2/ERF domain showed strong conservation across the analyzed ERF proteins, particularly around the YRG and RAYD-like motif regions, whereas the flanking regions were more variable. These results indicate that OsERF1 and OsERF94 retain the conserved AP2/ERF DNA-binding module typical of ERF transcription factors, while their divergent non-AP2 regions may contribute to functional differentiation.

### 2.2. Differential Stress-Responsive Expression Patterns of OsERF1 and OsERF94

**Figure 2:**
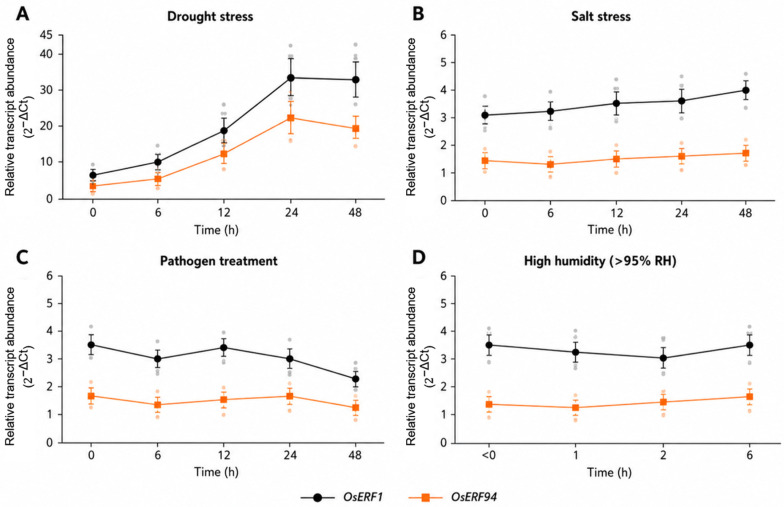
Time-course expression profiles of *OsERF1* and *OsERF94* under different environmental stress treatments. Relative transcript levels of *OsERF1* and *OsERF94* were determined by qRT-PCR following (**A**) drought treatment at 0, 6, 12, 24, and 48 h; (**B**) salt treatment at 0, 6, 12, 24, and 48 h; (**C**) pathogen treatment at 0, 6, 12, 24, and 48 h; and (**D**) high-humidity treatment (>95% relative humidity) at 0, 1, 2, and 6 h. Transcript levels were normalized to the internal reference gene and are presented as relative transcript abundance calculated using the 2^−ΔCt^ method. Dark-colored symbols indicate the mean expression levels, whereas the corresponding light-colored symbols represent individual biological replicates. Error bars indicate the mean ± standard deviation (SD) of three independent biological replicates. Statistical differences among sampling time points were analyzed separately for each gene using one-way ANOVA followed by Dunnett’s multiple-comparison test, with the corresponding 0 h sample used as the control.

To determine whether OsERF1 and OsERF94 respond to environmental conditions associated with stress adaptation and pre-harvest sprouting, their transcript levels were examined under drought, salt, pathogen, and high-humidity treatments (Figure 2). Under drought treatment, the transcript levels of both genes increased over time. *OsERF1* showed a pronounced increase at 12 h and reached its highest level at 24 h, which was maintained through 48 h. *OsERF94* also increased during drought treatment, although its induction was comparatively moderate at the later time points (Figure 2A). Under salt treatment, *OsERF1* expression gradually increased over the 48 h treatment period, whereas *OsERF94* exhibited only limited temporal variation (Figure 2B). Following pathogen treatment, both genes showed relatively modest changes compared with their responses to drought treatment. *OsERF1* exhibited minor fluctuations during the early treatment period followed by a decrease at 48 h, whereas *OsERF94* remained relatively stable throughout the treatment period (Figure 2C). Under high-humidity conditions, *OsERF1* showed only minor temporal variation, whereas *OsERF94* exhibited a gradual increase toward the later sampling points (Figure 2D). Collectively, these results demonstrate that *OsERF1* and *OsERF94* exhibit distinct treatment- and time-dependent expression patterns, with the strongest transcriptional response observed under drought conditions.

### 2.3. Generation and Validation of CRISPR/Cas9-Mediated Knockout Lines

**Figure 3:**
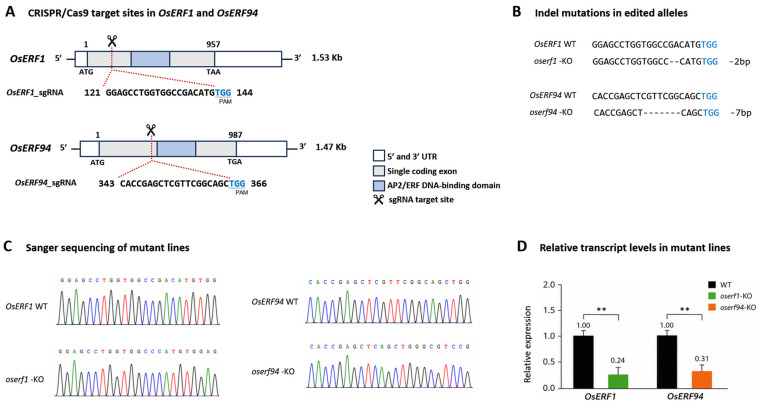
Generation and validation of CRISPR/Cas9-induced *oserf1*-KO and *oserf94*-KO mutant lines. (**A**) Schematic representation of the gene structures and CRISPR/Cas9 target sites of *OsERF1* and *OsERF94*. Both genes consist of a single coding exon. The sgRNA target sites were positioned in the 5′ coding region upstream of the AP2/ERF DNA-binding domain. The PAM sequences and predicted Cas9 cleavage sites are indicated. (**B**) Nucleotide sequence alignments of the predominant edited alleles identified by targeted amplicon deep sequencing of T_0_ plants. Dashes indicate deleted nucleotides. (**C**) Unedited Sanger sequencing chromatograms of WT plants and the selected homozygous T_2_ mutant lines across the corresponding CRISPR/Cas9 target regions. (**D**) Relative transcript abundance of *OsERF1* and *OsERF94* in WT plants and the corresponding homozygous T_2_ mutant lines, as determined by qRT-PCR. Transcript abundance was calculated using the 2^−ΔΔCt^ method, with the corresponding WT value set to 1.0. Data are presented as mean ± SD (*n* = 3 independent biological replicates). Asterisks indicate significant differences from WT (*p* < 0.01, Student’s *t*-test).

To investigate the functional roles of *OsERF1* and *OsERF94*, CRISPR/Cas9-mediated mutant lines were generated by targeting the single coding exon of each gene. The sgRNA target sites were positioned in the 5′ coding regions, upstream of the regions encoding the AP2/ERF DNA-binding domains (Figure 3A). Targeted amplicon deep sequencing of T_0_ plants identified a predominant 2 bp deletion at the *OsERF1* target site and a predominant 7 bp deletion at the *OsERF94* target site, accounting for 85.0% and 90.0% of the corresponding sequencing reads, respectively (Figure 3B; Table 1). Both deletions were predicted to shift the open reading frame and introduce premature termination codons upstream of the AP2/ERF DNA-binding domain. T_0_ plants carrying the desired edited alleles were self-pollinated, and their progeny were advanced through the T_1_ generation to the T_2_ generation. Sanger sequencing of individual T_2_ plants detected only the corresponding edited allele without a detectable WT sequence, confirming that the selected *oserf1*-KO and *oserf94*-KO lines were homozygous (Figure 3C). PCR screening with T-DNA-specific primers further identified homozygous T_2_ plants lacking detectable CRISPR/Cas9 T-DNA sequences. These homozygous, transgene-free T_2_ lines were used for subsequent phenotypic, molecular, and biochemical analyses. qRT-PCR analysis independently showed reduced steady-state transcript abundance of the corresponding target genes in the mutant lines compared with WT plants. The relative transcript abundance of *OsERF1* was 0.24 in the *oserf1*-KO mutant, whereas that of *OsERF94* was 0.31 in the *oserf94*-KO mutant when the corresponding WT value was set to 1.00 (Figure 3D). Collectively, the sequencing and PCR analyses confirmed the presence of homozygous frameshift mutations in the selected transgene-free T_2_ lines, whereas the qRT-PCR analysis showed reduced steady-state transcript abundance of the corresponding target genes.

### 2.4. OsERF1 and OsERF94 Differentially Regulate Pre-Harvest Sprouting

**Figure 4:**
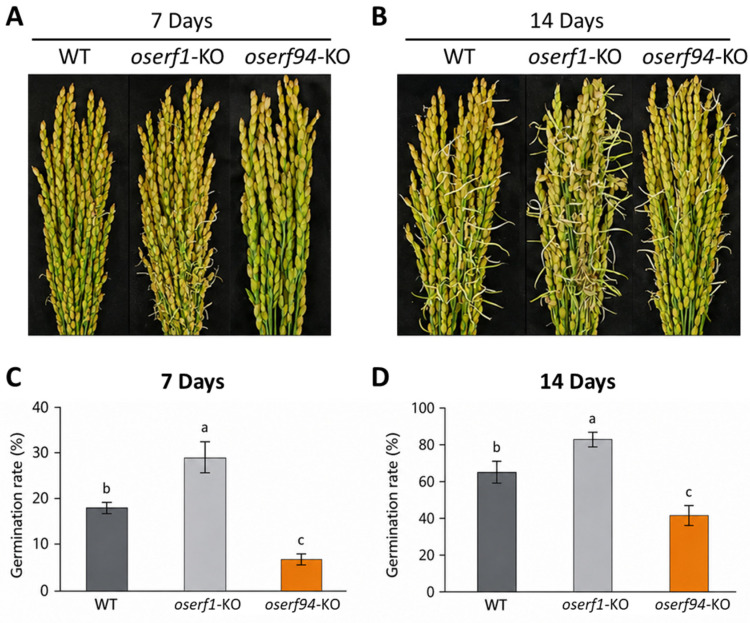
Sprouting phenotypes and sprouting rates of *oserf1*-KO and *oserf94*-KO mutant lines in a controlled detached-panicle PHS-inducing assay. Panicles of WT, *oserf1*-KO, and *oserf94*-KO plants were harvested at physiological maturity, approximately 40 days after heading, and immediately subjected to the controlled detached-panicle PHS-inducing assay without prior drying or storage. (**A**,**B**) Representative panicle images showing sprouting phenotypes at 7 and 14 days after the initiation of the assay, respectively. (**C**,**D**) Sprouting rates of WT, *oserf1*-KO, and *oserf94*-KO lines at 7 and 14 days, respectively. Data are presented as mean ± SD from three independent biological replicates. Different lowercase letters indicate significant differences among genotypes within each time point, as determined by one-way ANOVA followed by Tukey’s multiple-comparison test (*p* < 0.05).

To evaluate the roles of *OsERF1* and *OsERF94* in PHS, panicles of WT and mutant plants were harvested at physiological maturity, approximately 40 days after heading (DAH), and immediately subjected to a controlled detached-panicle PHS-inducing assay without prior drying or storage. Representative panicle images showed that the *oserf1*-KO mutant exhibited enhanced sprouting compared with WT at 7 days after the initiation of the assay, whereas the *oserf94*-KO mutant displayed reduced sprouting even after 14 days under high-humidity conditions (Figure 4A,B). Quantitative analysis of sprouting rates further supported these contrasting phenotypes. At 7 days, the sprouting rate was significantly higher in *oserf1*-KO than in WT, whereas *oserf94*-KO showed a significantly lower sprouting rate (Figure 4C; Table 2). At 14 days, *oserf1*-KO maintained a higher sprouting rate than WT, whereas the sprouting rate of *oserf94*-KO remained lower than that of WT despite the extended incubation period (Figure 4D; Table 2). These results indicate that loss of *OsERF1* promotes PHS-associated sprouting, whereas loss of *OsERF94* suppresses sprouting under the controlled detached-panicle PHS-inducing conditions. Because sprouting of freshly harvested grains under standardized high-humidity conditions is inversely associated with seed dormancy, the increased sprouting observed in *oserf1*-KO suggests reduced dormancy. By contrast, the reduced sprouting observed in *oserf94*-KO may reflect enhanced dormancy and/or reduced metabolic competence for germination. Together, these findings support a differential regulatory model in which *OsERF1* acts as a negative regulator of PHS-associated sprouting, whereas *OsERF94* functions as a positive regulator.

### 2.5. OsERF1 Regulates ABA-Dependent Dormancy, Whereas OsERF94 Controls Metabolic Activation During Germination

**Figure 5:**
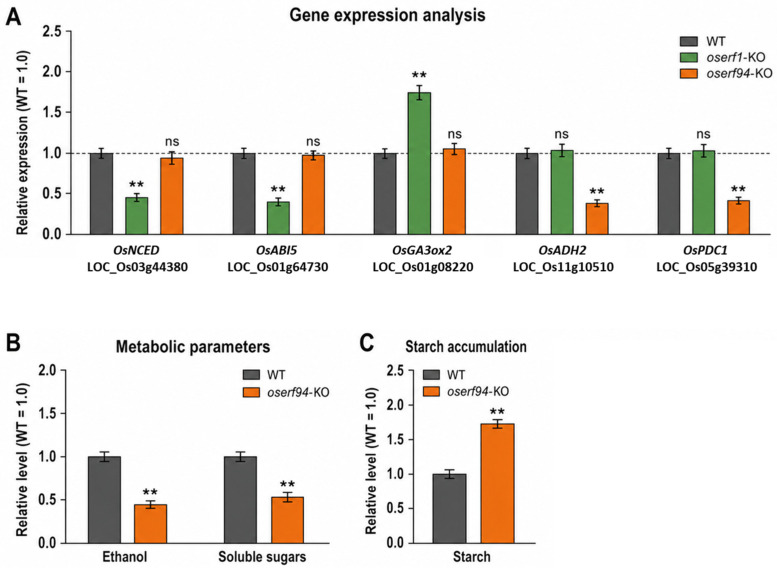
Distinct molecular pathways associated with *OsERF1* and *OsERF94* during dormancy release and germination. (**A**) Relative expression levels of hormone- and metabolism-related genes in WT, *oserf1-KO*, and *oserf94-KO* lines. The dashed horizontal line indicates the WT-normalized reference level of 1.0. (**B**) Relative levels of ethanol and soluble sugars in WT and *oserf94-KO* seeds. (**C**) Relative starch accumulation in WT and *oserf94-KO* seeds. Values were normalized to WT, which was set to 1.0. Data are presented as mean ± SD. Asterisks indicate significant differences compared with WT (** *p* < 0.01); ns, not significant.

To investigate the molecular basis underlying the contrasting germination phenotypes of *oserf1-KO* and *oserf94-KO* lines, the expression of hormone- and metabolism-related genes was examined together with key metabolic parameters. The analyzed genes included ABA-related markers, GA-associated genes, and hypoxia-responsive genes involved in fermentative metabolism (Figure 5A; Table 3). In the *oserf1-KO* line, the expression levels of ABA biosynthesis and signaling-related genes, including *NCED* and *ABI5*, were significantly reduced compared with WT, whereas the expression of a GA-related gene was increased (Figure 5A). In contrast, these hormone-related genes showed little or no significant change in the *oserf94-KO* line. These expression patterns suggest that loss of *OsERF1* disrupts ABA-associated dormancy maintenance and shifts the hormonal balance toward germination-promoting regulation. By contrast, *oserf94-KO* exhibited a distinct molecular profile. Although ABA- and GA-related gene expression was largely unchanged, the hypoxia-responsive genes *ADH* and *PDC* were markedly downregulated in *oserf94-KO* compared with WT (Figure 5A). Consistent with this reduction in fermentative gene expression, ethanol and soluble sugar levels were significantly decreased in *oserf94-KO*, whereas starch accumulation was increased (Figure 5B,C; Table 3). These results indicate that loss of *OsERF94* impairs metabolic activation during germination, particularly carbohydrate mobilization and anaerobic/fermentative metabolism. Together, these findings suggest that *OsERF1* and *OsERF94* regulate pre-harvest sprouting through distinct molecular mechanisms. *OsERF1* appears to modulate ABA/GA-associated dormancy control, whereas *OsERF94* is more closely associated with the metabolic transition required for germination, including hypoxia-responsive gene expression and starch-to-sugar mobilization.

## 3. Discussion

PHS is a complex agronomic trait in rice that occurs when mature seeds germinate on the mother plant before harvest, particularly under high-humidity or rainfall-prone conditions [1]. In the present study, CRISPR/Cas9-induced frameshift mutations in two AP2/ERF transcription factor genes, *OsERF1* and *OsERF94*, produced contrasting effects on PHS-associated germination. Disruption of *OsERF1* promoted germination and increased PHS susceptibility, whereas disruption of *OsERF94* suppressed germination and reduced PHS (Figure 3 and Figure 4; Table 1 and Table 2). ERF proteins contain a conserved AP2/ERF DNA-binding domain and are widely involved in plant development, hormone responses, and environmental stress adaptation [20,21,27,28].

Both OsERF1 and OsERF94 retained the conserved YRG and RAYD elements within their AP2/ERF domains (Figure 1 and Figure S1), consistent with the characteristic structural features of AP2/ERF-family proteins. However, their divergent N- and C-terminal regions may contribute to differences in regulatory activity, target specificity, or protein–protein interactions, thereby explaining their non-redundant roles in PHS-associated responses. Targeted amplicon deep sequencing of T_0_ plants and Sanger sequencing of T_2_ progeny confirmed the presence and stable inheritance of the corresponding frameshift mutations. qRT-PCR independently showed reduced steady-state transcript abundance of *OsERF1* and *OsERF94* in their respective mutant lines (Figure 3). Because the deletions were located within the coding regions rather than the promoter regions, the reduced transcript abundance is unlikely to result from disruption of transcription initiation. Instead, it may reflect reduced stability of the frameshifted transcripts, potentially through nonsense-mediated mRNA decay or another RNA-surveillance mechanism triggered by the predicted premature termination codons. However, this possibility was not directly examined, and the underlying mechanism remains unresolved.

The phenotype of *oserf1*-KO suggests that OsERF1 is associated with negative regulation of PHS-related germination. Seed dormancy and germination are controlled by a complex regulatory network in which hormonal balance plays a central role [29]. ABA promotes dormancy maintenance, whereas dormancy release and germination require attenuation of ABA-mediated repression [2,3]. In this study, *oserf1*-KO showed an increased germination percentage, a reduced dormancy index, and an elevated PHS rate compared with WT plants (Figure 4; Table 2). These phenotypes were accompanied by reduced ABA content and decreased transcript abundance of the ABA-related genes *NCED* and *ABI5* (Figure 5A; Table 3). Because NCED is associated with ABA biosynthesis and ABI5 is an important ABA-signaling component involved in the repression of germination, their reduced expression is consistent with weakened ABA-associated dormancy maintenance. In parallel, *oserf1*-KO showed increased GA content and elevated expression of the examined GA-related gene. Because ABA and GA act antagonistically during dormancy release and germination [11,12,30,31,32], these results suggest that disruption of OsERF1 alters ABA/GA-associated dormancy regulation and facilitates premature germination under high-humidity conditions. Nevertheless, direct regulation of these hormone-related genes by OsERF1 was not demonstrated. This interpretation is consistent with broader models in which dormancy release is governed by coordinated changes in ABA metabolism, GA signaling, and reciprocal hormonal interactions [33,34]. In contrast, the *oserf94*-KO phenotype indicates that OsERF94 is associated with PHS regulation through a mechanism distinct from ABA/GA-mediated dormancy control. The *oserf94*-KO line exhibited a reduced germination percentage, an increased dormancy index, and a lower PHS rate than WT plants (Figure 4; Table 2), whereas its ABA and GA levels were not significantly altered (Table 3). Instead, *oserf94*-KO showed reduced transcript abundance of the hypoxia-responsive genes *ADH* and *PDC*, together with reduced ethanol and soluble sugar levels and increased starch accumulation (Figure 5 and Figure S2; Table 3). These findings suggest that the reduced germination of *oserf94*-KO may be associated with impaired metabolic readiness rather than strengthened hormone-mediated dormancy. The proposed metabolic role of OsERF94 is particularly relevant under PHS-inducing high-humidity conditions. During seed imbibition and germination, oxygen availability within seed tissues may become locally restricted, requiring metabolic adaptation to oxygen-limited conditions. Plants respond to oxygen limitation by activating glycolytic and fermentative pathways to maintain energy production [16], and rice seeds rely on metabolic adaptation during germination under oxygen-deficient environments [19]. In rice, genotypic differences in underwater germination are closely associated with differential expression of alcoholic fermentation enzymes [35]. Soluble sugars provide substrates for glycolysis, whereas fermentation regenerates NAD+ and supports continued ATP production. The reduced expression of *ADH* and *PDC* in *oserf94*-KO, together with decreased ethanol accumulation, is consistent with reduced fermentative activity (Figure 5A,B; Table 3). The increased starch content and reduced soluble sugar content further suggest inefficient conversion of stored starch into metabolically available sugars (Figure 5B,C and Figure S2; Table 3). This interpretation is consistent with evidence that starch availability and metabolism are required for hypoxia-responsive gene regulation and survival [36], and that alcohol dehydrogenase and pyruvate decarboxylase contribute to plant growth and metabolic acclimation [37]. Because fermentation depends on glycolytic carbon flux, restricted starch-to-sugar mobilization could limit the energy supply required for germination. However, direct regulation of *ADH*, *PDC*, or carbohydrate-mobilization genes by OsERF94 was not demonstrated.

**Figure 6:**
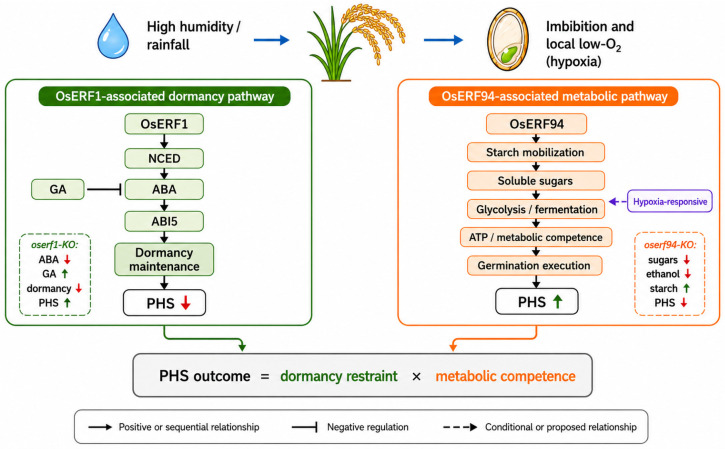
Proposed model for OsERF1- and OsERF94-mediated regulation of pre-harvest sprouting in rice. High humidity or rainfall promotes seed imbibition and creates a local low-O_2_ environment. OsERF1 is proposed to maintain dormancy through the NCED–ABA–ABI5 pathway, with GA acting antagonistically. Loss of OsERF1 reduces ABA-associated dormancy restraint and increases PHS susceptibility. In contrast, OsERF94 is proposed to promote starch mobilization, soluble sugar production, glycolysis and fermentation, ATP production, and metabolic competence required for germination. Loss of OsERF94 reduces sugar and ethanol accumulation, increases starch retention, and suppresses PHS. Thus, PHS outcome is determined by the balance between dormancy restraint and metabolic competence. Blue arrows indicate environmental progression, whereas the green and orange pathways represent OsERF1-associated dormancy regulation and OsERF94-associated metabolic regulation, respectively. The purple arrow indicates hypoxia-responsive regulation. Solid arrows indicate positive or sequential relationships, blunt-ended lines indicate negative regulation, and dashed arrows indicate conditional or proposed relationships. Up arrows indicate increase and down arrows indicate decrease.

Together, the contrasting mutant phenotypes support a dual-layer model of PHS-associated germination (Figure 6). The first layer involves hormone-dependent dormancy control and is associated primarily with OsERF1. When OsERF1 is disrupted, ABA-associated dormancy maintenance is weakened, GA-associated activity is enhanced, and seeds become more susceptible to premature germination. The second layer involves metabolism-dependent germination capacity and is associated primarily with OsERF94. When OsERF94 is disrupted, activation of hypoxia-responsive fermentation and carbohydrate mobilization is reduced, thereby limiting germination even under PHS-inducing conditions. This framework suggests that PHS susceptibility depends not only on dormancy release but also on whether seeds possess sufficient metabolic capacity to complete germination.

Several limitations should be considered when interpreting these findings. Direct binding of OsERF1 and OsERF94 proteins to the promoters of the genes examined in this study was not experimentally validated. Future studies using electrophoretic mobility-shift assays, yeast one-hybrid assays, dual-luciferase reporter assays, or ChIP-qPCR will be required to determine whether the observed downstream genes are direct targets. Genetic complementation and overexpression analyses would also strengthen the causal interpretation of the mutant phenotypes. In addition, PHS-associated sprouting was evaluated using a controlled detached-panicle PHS-inducing assay. This assay provided standardized temperature and humidity conditions for comparing intrinsic genotype-dependent panicle and grain responses while minimizing environmental variation among plants. Nevertheless, detachment of the panicles may alter physiological interactions between the developing grains and the mother plant, including maternal regulation that may contribute to the suppression of premature germination. Therefore, the controlled detached-panicle PHS-inducing assay does not fully reproduce PHS occurring on intact mother plants or the complex and fluctuating conditions associated with natural rainfall in the field. Further validation using intact-plant high-humidity treatments and field-based rainfall conditions will be required to confirm the physiological and agronomic relevance of the observed mutant phenotypes.

## 4. Materials and Methods

### 4.1. Plant Materials and Growth Conditions

Rice (*Oryza sativa* L. cv. Dongjin) was used as the wild-type background in this study. CRISPR/Cas9-mediated knockout lines targeting *OsERF1* and *OsERF94* were generated and selected as described below. All experiments, beginning with rice transformation in March 2023 and followed by plant cultivation, genotyping, and phenotypic analyses, were conducted through October 2025 in the Plant Breeding Laboratory at Hankyong National University, Anseong, Republic of Korea. Plants were cultivated in a controlled greenhouse under standard growth conditions consisting of 28 °C/25 °C day/night temperatures, 60–70% relative humidity, and a 12 h light/12 h dark photoperiod. Plants were grown in soil and maintained by regular irrigation and fertilization according to standard rice cultivation practices. For experiments using freshly harvested panicles or seeds, panicles were collected at physiological maturity, approximately 40 DAH, and used immediately without prior drying or storage. For experiments requiring stored seed material, harvested panicles were air-dried at room temperature for 5–7 days and subsequently stored under dry conditions until use. For phenotypic, molecular, physiological, and biochemical analyses, samples were collected from plants grown under the same environmental conditions and at comparable developmental stages to minimize variation caused by growth stage or cultivation conditions.

### 4.2. CRISPR/Cas9 Vector Construction and Rice Transformation

Target-specific 20-nucleotide protospacer sequences were selected for *OsERF1* (Os04g0546800; LOC_Os04g46220) and *OsERF94* (Os04g0547600; LOC_Os04g46250). Because both genes consist of a single coding exon, candidate protospacers were positioned in the 5′ coding region upstream of the region encoding the AP2/ERF DNA-binding domain to increase the likelihood of generating frameshift mutations. Candidate sequences were selected using Cas-Designer from CRISPR RGEN Tools (http://www.rgenome.net/cas-designer/, accessed on 16 May 2023) based on the presence of a suitable PAM, predicted target specificity, GC content, out-of-frame score, position within the coding region, and minimal predicted off-target potential [38]. Potential off-target sites were further evaluated using Cas-OFFinder from the same platform (http://www.rgenome.net/cas-offinder/, accessed on 16 May 2023) [39]. The selected protospacer and corresponding PAM sequences are listed in Supplementary Table S1. The standard sgRNA scaffold was already encoded in the binary CRISPR/Cas9 vector and was not independently designed or modified in this study. Only oligonucleotides corresponding to the target-specific 20-nucleotide spacer sequences were synthesized by Macrogen Inc. (Seoul, Republic of Korea) and cloned upstream of the vector-derived sgRNA scaffold. sgRNA expression was driven by the rice OsU3 promoter, whereas Cas9 expression was controlled by the maize ubiquitin promoter. The resulting constructs were verified by restriction enzyme digestion and Sanger sequencing using vector-specific primers and introduced into *Agrobacterium tumefaciens* strain EHA105 by electroporation. Rice transformation was performed using embryogenic calli derived from mature seeds according to an established *Agrobacterium*-mediated transformation procedure with minor modifications [40,41]. Transformed calli were selected on phosphinothricin-containing medium, regenerated under tissue-culture conditions, transferred to soil, and screened by PCR using vector-specific primers.

### 4.3. Genotyping, Segregation, and Mutation Analysis

Genomic DNA was extracted from young leaves of wild-type and putative mutant plants using a modified CTAB method [42]. Briefly, fresh leaf tissue was ground in liquid nitrogen and incubated in CTAB extraction buffer. DNA quality and concentration were assessed using a NanoDrop 2000 spectrophotometer (Thermo Fisher Scientific, Waltham, MA, USA) and by agarose gel electrophoresis. Target regions flanking the sgRNA sites of *OsERF1* and *OsERF94* were amplified by PCR using gene-specific primers listed in Supplementary Table S1. PCR amplification was performed using a T100 Thermal Cycler (Bio-Rad Laboratories, Hercules, CA, USA). CRISPR/Cas9-induced mutations in primary transgenic plants of the T_0_ generation were initially analyzed by targeted amplicon deep sequencing. The resulting sequencing reads were analyzed using Cas-Analyzer (http://www.rgenome.net/cas-analyzer/, accessed on 17 September 2024) to identify edited alleles and determine their relative frequencies. T_0_ plants carrying the desired edited alleles were self-pollinated, and their progeny were advanced through the T_1_ generation to the T_2_ generation. For genotyping of individual T_2_ plants, target regions encompassing the CRISPR/Cas9 cleavage sites were amplified by PCR. The purified PCR products were subjected to Sanger sequencing by Macrogen Inc. (Seoul, Republic of Korea), and the resulting chromatograms were analyzed using SnapGene software version 7.2.1 (GSL Biotech LLC, San Diego, CA, USA). Edited sequences were compared with the corresponding wild-type genomic sequences to determine the type and size of each indel. T_2_ plants were classified as homozygous when the chromatograms showed a single edited sequence without detectable overlapping peaks corresponding to the wild-type allele at the target site.

### 4.4. RNA Extraction and Quantitative Real-Time PCR (qRT-PCR)

Total RNA was extracted from rice tissues using TRIzol reagent (Invitrogen, Thermo Fisher Scientific, Waltham, MA, USA) according to the manufacturer’s instructions. Residual genomic DNA was removed by treatment with DNase I. RNA concentration and purity were measured using a NanoDrop 2000 spectrophotometer (Thermo Fisher Scientific), and RNA integrity was evaluated by agarose gel electrophoresis. First-strand cDNA was synthesized from total RNA using the PrimeScript RT Reagent Kit (Takara Bio Inc., Shiga, Japan) according to the manufacturer’s protocol. Quantitative real-time PCR was performed using TB Green Premix Ex Taq II (Takara Bio Inc.) on a CFX96 Real-Time PCR Detection System (Bio-Rad Laboratories, Hercules, CA, USA). Gene-specific primers used for qRT-PCR are listed in Supplementary Table S1. *OsActin* was used as the internal reference gene. For the time-course stress-expression analyses, relative transcript abundance was calculated using the 2^−ΔCt^ method, where ΔCt was defined as Ct_target − Ct_*OsActin*. For comparisons between mutant and wild-type plants, relative expression levels were calculated using the 2^−ΔΔCt^ method [43], with the wild-type sample used as the calibrator. Each analysis included three independent biological replicates, and each biological replicate was analyzed using three technical replicates.

### 4.5. Stress Treatments and Expression Analysis

To examine the stress-responsive expression patterns of *OsERF1* and *OsERF94*, three-week-old wild-type Dongjin rice seedlings were subjected to simulated drought, salt, and pathogen treatments. For simulated drought treatment, seedlings were transferred to a nutrient solution containing 20% (*w*/*v*) polyethylene glycol 6000 (PEG 6000), whereas control seedlings were maintained in the same nutrient solution without PEG 6000. For salt treatment, seedlings were exposed to 150 mM NaCl, whereas untreated seedlings maintained in the same nutrient solution were used as controls. Leaf samples were collected immediately before treatment (0 h) and at 6, 12, 24, and 48 h after the initiation of each treatment. For pathogen treatment, three-week-old seedlings were spray-inoculated with a conidial suspension of *Magnaporthe oryzae* isolate KJ201 at a concentration of 1 × 10^5^ conidia mL^−1^ containing 0.02% (*v*/*v*) Tween 20. Mock-treated seedlings sprayed with sterile distilled water containing 0.02% (*v*/*v*) Tween 20 were used as controls. After inoculation, the seedlings were maintained at 25 °C in darkness and at >95% relative humidity for 24 h and were subsequently returned to the standard growth conditions described above. Leaf samples were collected immediately before inoculation (0 h) and at 6, 12, 24, and 48 h after inoculation. KJ201 is a documented rice-infecting *M. oryzae* isolate. For high-humidity treatment, panicles were harvested at physiological maturity, approximately 40 DAH, and immediately incubated in a controlled growth chamber maintained at >95% relative humidity and 28 °C. Spikelets were collected immediately before treatment (0 h) and at 1, 2, and 6 h after the initiation of the high-humidity treatment. All collected samples were immediately frozen in liquid nitrogen and stored at −80 °C until RNA extraction. Transcript abundance was calculated using the 2^−ΔCt^ method. Statistical comparisons among sampling time points were performed separately for each gene, with the corresponding 0 h sample used as the control.

### 4.6. Controlled Detached-Panicle Pre-Harvest Sprouting Assay

A controlled detached-panicle PHS-inducing assay was conducted to compare intrinsic genotype-dependent panicle and grain responses under standardized high-humidity conditions. Panicles of WT, *oserf1*-KO, and *oserf94*-KO plants were harvested at physiological maturity, approximately 40 days after heading (DAH), and immediately used without prior drying or storage. The detached panicles were incubated in a controlled chamber maintained at 95–100% relative humidity and 28 °C for 14 days. Sprouted grains were visually identified and counted at 7 and 14 days after the initiation of the assay. A grain was considered sprouted when visible emergence of the radicle or coleoptile was observed. The sprouting rate was calculated as follows: Sprouting rate (%) = (number of sprouted grains/total number of grains examined) × 100. Each genotype included three independent biological replicates, with panicles collected from independently grown plants. The controlled detached-panicle PHS-inducing assay was selected to provide uniform temperature and humidity conditions, thereby allowing direct comparison of intrinsic genotype-dependent panicle and grain responses while minimizing environmental variation among plants.

### 4.7. Hormone Quantification

Seed tissues were collected from WT, oserf1-KO, and oserf94-KO panicles at 7 days after initiation of the controlled detached-panicle PHS-inducing assay described in Section 4.6. The samples were immediately frozen in liquid nitrogen and stored at −80 °C until analysis. Endogenous abscisic acid (ABA) and gibberellic acid (GA) levels were quantified using a Plant Abscisic Acid ELISA Kit (Bioassay Technology Laboratory, Shanghai, China; Cat. No. E0002Pl) and a Plant Gibberellic Acid ELISA Kit (Bioassay Technology Laboratory, Shanghai, China; Cat. No. E0000Pl), respectively, according to the manufacturer’s instructions. Absorbance was measured using a microplate reader, and hormone concentrations were calculated from the corresponding standard curves. Hormone levels were normalized to fresh weight and expressed as ng g^−1^ FW. Three independent biological replicates were analyzed for each genotype.

### 4.8. Metabolite Analysis

#### 4.8.1. Ethanol Measurement

Ethanol content was determined using an enzymatic ethanol assay kit according to the manufacturer’s instructions. Briefly, seed tissues were homogenized in extraction buffer and centrifuged, and the supernatant was used for ethanol quantification. Absorbance was measured using a UV-1800 spectrophotometer (Shimadzu, Kyoto, Japan). Ethanol concentration was calculated using a standard curve and expressed on a fresh weight basis.

#### 4.8.2. Soluble Sugar Quantification

Soluble sugars were extracted from seed tissues using 80% ethanol. Total soluble sugar content was quantified using the anthrone method with glucose as the standard [44]. Absorbance was measured at 620 nm using a UV-1800 spectrophotometer (Shimadzu, Kyoto, Japan). Soluble sugar content was expressed as mg g^−1^ FW.

#### 4.8.3. Starch Measurement

Starch content was determined by enzymatic hydrolysis followed by glucose quantification. After removal of soluble sugars, the remaining pellet was dried and hydrolyzed using α-amylase and amyloglucosidase. Released glucose was quantified using a glucose-based colorimetric assay. Starch content was calculated based on glucose equivalents and expressed on a fresh weight basis. All metabolite measurements were performed with at least three biological replicates.

### 4.9. Statistical Analysis

All experiments were conducted with at least three biological replicates unless otherwise stated. Data are presented as the mean ± standard deviation (SD). Statistical analyses were performed using IBM SPSS Statistics version 26.0 (IBM Corp., Armonk, NY, USA) and GraphPad Prism version 9.0 (GraphPad Software, San Diego, CA, USA). Differences between two groups were analyzed using Student’s *t*-test. For comparisons among multiple genotypes, one-way analysis of variance (ANOVA) followed by Tukey’s honestly significant difference test was performed. For time-course expression analyses, statistical differences among sampling time points were evaluated separately for each gene and treatment using one-way ANOVA followed by Dunnett’s multiple-comparison test, with the corresponding pretreatment sample used as the control. Differences were considered statistically significant at *p* < 0.05.

## 5. Conclusions

This study demonstrates that *OsERF1* and *OsERF94* regulate PHS-associated germination through distinct mechanisms in rice. *OsERF1* functions primarily in ABA/GA-associated dormancy control, and loss of *OsERF1* promotes premature germination under high-humidity conditions. In contrast, *OsERF94* contributes to metabolic activation during germination by supporting hypoxia-responsive gene expression, fermentative metabolism, and carbohydrate mobilization. Loss of *OsERF94* impairs these metabolic processes and suppresses PHS-associated germination. Together, these findings suggest that PHS susceptibility is controlled by both hormone-dependent dormancy regulation and metabolism-dependent germination capacity, providing potential targets for improving PHS resistance in rice.

## Figures and Tables

**Table 1:** Summary of CRISPR/Cas9-induced mutations in *OsERF1* and *OsERF94*.

Line	Predominant Edited Allele Frequency in T_0_ Plants (%)	Indel Size (bp)	Mutation Type	Predicted Consequence	T_2_ Genotype
*oserf1*-KO	85.0	−2	Deletion	Frameshift and premature termination codon	Homozygous
*oserf94*-KO	90.0	−7	Deletion	Frameshift and premature termination codon	Homozygous

**Table 2:** PHS-associated sprouting and dormancy traits of WT and mutant lines at 14 days after treatment.

Genotype	Sprouting (%)	Dormancy Index	PHS Rate (%)
WT	65.3 ± 3.2 b	0.42 ± 0.03 b	18.7 ± 2.1 b
*oserf1*-KO	84.6 ± 2.8 c	0.25 ± 0.02 a	36.5 ± 3.4 c
*oserf94*-KO	42.1 ± 3.5 a	0.61 ± 0.04 c	9.3 ± 1.8 a

Values are presented as mean ± SD from at least three biological replicates. Significant differences compared to WT are indicated by different lowercase letters (*p* < 0.05, Tukey’s test).

**Table 3:** Hormone levels and metabolic changes in WT and mutant lines under PHS-inducing conditions.

Genotype	ABA (ng g^−1^ FW)	GA (ng g^−1^ FW)	Ethanol (µmol g^−1^ FW)	Soluble Sugars (mg g^−1^ FW)	Starch (mg g^−1^ FW)
WT	125.4 ± 8.6	42.7 ± 3.5	0.52 ± 0.04	10.8 ± 0.9	9.8 ± 0.8
*oserf1*-KO	78.6 ± 6.2 **	65.3 ± 4.1 **	0.55 ± 0.05 ns	11.2 ± 1.1 ns	9.5 ± 0.7 ns
*oserf94*-KO	118.2 ± 7.4 ns	45.1 ± 3.8 ns	0.31 ± 0.03 **	8.2 ± 0.7 **	12.6 ± 1.1 **

** Values are presented as mean ± SD (*n* ≥ 3 biological replicates). Asterisks indicate significant differences compared to wild-type plants (*p* < 0.01), and “ns” indicates no significant difference. FW, fresh weight.

## Data Availability

The original contributions presented in this study are included in the article/Supplementary Materials. Further inquiries can be directed to the corresponding authors.

## Supplementary Materials

The following supporting information can be downloaded at: https://www.mdpi.com/article/10.3390/ijms27135915/s1.
