# Supplementary material for: Differential Regulation of Pre-Harvest Sprouting by OsERF1 and OsERF94 Through Hormone Signaling and Metabolic Reprogramming in Rice
Source: Int J Mol Sci. 2026 Jun 30;27(13):5915. doi: 10.3390/ijms27135915 (PMC13362466; doi:10.3390/ijms27135915)
Supplement: Supplementary file 1 [file ijms-27-05915-s001.zip › ijms-4374275-supplementary.pdf]

Supplementary Data

Differential Regulation of Pre-Harvest Sprouting by *OsERF1* and *OsERF94* Through Hormone Signaling and Metabolic Reprogramming in Rice

Yu-Jin Jung [1,2], Jong-Hee Kim [1], Jin-Young Kim [1], Jiyun Go [3], Hak-Soo Kim [1], Sang-Mun Jung [1] and Kwon Kyoo Kang [1,2,*]

[1] Division of Horticultural Biotechnology, Hankyong National University, Anseong 17579, Republic of Korea; yuyu1216@hknu.ac.kr (Y.-J.J.); jonghee014@hknu.ac.kr (J.-H.K.); zino@hknu.ac.kr (J.-Y.K.); haksoolove@naver.com (H.-S.K.); mocgidan@hanmail.net (S.-M.J.)
[2] Institute of Genetic Engineering, Hankyong National University, Anseong 17579, Republic of Korea
[3] Department of Bio-Environmental Chemistry, College of Agriculture and Life Sciences, Chungnam National University, Daejeon 34134, Republic of Korea; jy.go2369@gmail.com
* Correspondence: kykang@hknu.ac.kr; Tel.: +82-31-670-5104

Contents

Supplementary Figure S1. Full-length sequence alignment of *OsERF1*, *OsERF94*, and representative ERF family proteins.

Supplementary Figure S2. Raw data distribution of metabolite contents.

Supplementary Table S1. List of oligonucleotides and primers used in this study.

```
OsERF1        1    MTARSMLRNHPEASVLDTIRQHLLEEPRGGGGGEAAEASFGSLVADMWSDSLPFRDDDADDMVVFGAMRDAFSCGWLPDG   80
OsERF94       1    ---MDFHHGDADDFALEFIREHLL-------GVDGATATATEPADLEVVEPAAAYPPMSWQEQRQQEQHGCHVELTDEHL   70
GhERF1        1    -----MPATFEESTTLEFIRQHLL-------GDFATVDAFINTLDFGLSHLQ----------------------------   40
AtERF1        1    ----MDPFLIQSPFSG------------------------------------------FSPEYSIGSSPDSFSSSSSNNY   34
TaPIE1        1    ---MSDPSASSTASSSPTSSSTSP-------RLTTGVVNFLARRAVTTQQNHHLPAAEAFPLHSPGSSTGSADSAPWHHR   70
SlERF1             --------------------------------------------------------------------------------
ZmERF1-like   1    MCGGAILAELREPAPRRLTERDIWQ---------------------------------QKKKPKRGGAGGRRSFAAEDDE   47
SbERF039           --------------------------------------------------------------------------------

                                                                                         YGD motif
OsERF1       81    VFAEVKPEPLLSPDSSSYDGSSCCFGFADVSEPVTPSDAASGAAEAAAAAAAATAEHGKEEEAAAAVARGKHYRGVRQRP   160
OsERF94      71    ESAPAAEAAAAFRTAPAQPAAEVMIKFGGEPSPVRPSSSLT----ISLPPSSFGSWASAAAPAAAAVEDFRKYRGVRQRP   146
GhERF1       41    ---------------PQSHQLPEIFTHGVEPAPITKEP---------------------------FCEEKRHYRGVRRRP   78
AtERF1       35    S-------LPFNENDSEEMFLYGLIEQSTQQTYIDSDSQ------------DLPIKSVSSRKS------EKSYRGVRRRP   89
TaPIE1       71    APATPPPVLPFDPSDADEMLLLDMLSQHHEVQHAAAAPT------------TAPAKQEADEEGKVALGVGRAFRGVRKRP   138
SlERF1        1    -----------------------------------------------------------------MARAQQRYRGVRQRH   15
ZmERF1-like  48    DFEADFEDFEADSGDSDLELGEGADDDVIEIKPFAAKSTFS----RDGLSTMTTAGYDAPAARLAKRKRKNQYRGIRQRP   123
SbERF039      1    ----------MDMDDAQEPASPTSPSPSSSSSSSSSSSSVG-----------------PKKRARKDGSRHPTYRGVRMRS   53

                                                RAYD-like motif
OsERF1      161    WGKFAAEIRDPAKNGARVWLGTFDTAEDAALAYDRAAYRMRGS-RALLNFPLRIGSEIAAAAAAAAAAAAGDKRPSPEPA   239
OsERF94     147    WGKFAAEIRDPKRRGSRVWLGTYDTPVEAARAYDRAAFRMRGA-KAILNFPNEVGTRGAELWAPPPPPPAHSAAASTTNK   225
GhERF1       79    WGKFAAEIRDPNRKGIRVWLGTYDSDVDAAKAYDCAAFKMRGQ-KAILNFPLEAGEG--------SQPPAVTTGRKRRRE   149
AtERF1       90    WGKFAAEIRDSTRNGIRVWLGTFESAEEAALAYDQAAFSMRGS-SAILNFSAERVQESLS---EIKYTYEDGCSPVVALK   165
TaPIE1      139    WGKFAAEIRDSTRNGVRVWLGTFDSPEAAALAYDQAAFAIRGG-AAVLNFPADQVRRSLEGAADDACGRAHGLSPVLALK   217
SlERF1       16    WGSWVSEIR-HPLLKTRIWLGTFETAEDAARAYDEAARLMCGP-RARTNFPYNPNMPQTSSSKLLSTTLTAKLHKCYMAS   93
ZmERF1-like 124    WGKWAAEIRDPQK-GVRVWLGTFNSPEEAARAYDAEARRIRGK-KAKVNFPDAPAVGQKCRSSSASAKALKSCVEQKPIV   201
SbERF039     54    WGKWVSEIREPRK-KSRIWLGTFATAEMAARAHDVAALAIKGARDAHLNFPSQAHLLPRPASAAPKDVQAAAALAAAADF   132

OsERF1      240    TSES------------SFSSSSSCTTTTTSSSTSSSGSPKRRKRGEAAAASMSMPLVPPPSQLNWPVQAWYPAAAPVEQV   307
OsERF94     226    RKRQ------------PSEDPDDGVEVIGVVSKAVKTEAPTSNSSSLSSSLTSRDTTPATSSAGAEHAGAAAESSPATPS   293
GhERF1      150    KRVW------------LPESDITSPGSSEMAWEVKEEEGELDDDDRNGLSLLTRKRVIVTC-------------------   198
AtERF1      166    RKHS------------MRRRMTNKKTKDSDFDHRSVKLDNVVVFEDLGEQYLEELLGSSENSGTW---------------   218
TaPIE1      218    RRHC------------MRSRKAAAAGRVAKAATRGVRPGCVMELEDLGAEYLEQLLGASEDTASMSSWCWSHQSV-----   280
SlERF1       94    LQMTKTSPQGQKLAKNATNVQESVINSYKMKQQMLVPKPSVLLTHHDHHEEAKVVNLGVGVIRKVEDQVLEGIPQFVKPL   173
ZmERF1-like 202    KTDMNILANTNAPFYQSVNYASNNPFVPAMNSTVSFEDPIMNLHSDQGSNSLGCSDLGWENDTKTPDITSIAPIPTIAEG   281
SbERF039    133    PPLP------------SSCPCPGEGAGNAKSPESSSSDDASASAASPPPPPPPPPPAPQEDAEPNPDDALFDLPDLLLDL   200

OsERF1      308    AITPRVEQLVI---------------------------------------------------------------------   318
OsERF94     294    SWSWEQYWEALLGGLPPLSPLSPHPALGFPQLTVN---------------------------------------------   328
GhERF1             --------------------------------------------------------------------------------
AtERF1             --------------------------------------------------------------------------------
TaPIE1             --------------------------------------------------------------------------------
SlERF1      174    EDDHIEQMIEELLDYGSIELCSNVVPSHQIQ-------------------------------------------------   204
ZmERF1-like 282    DESVFVNSNSNSSMVPPVLENNAVDLTDGLTDLESYMRFLMDGGASDSIDSLLNLDGSQDLGSNMDLWTFDDMPIAGDFF   361
SbERF039    201    RCCGPSSWAVADDDVAGGGAFRLIEEPLLWEY------------------------------------------------   232
```

Supplementary Figure S1. Full-length sequence alignment of *OsERF1*, *OsERF94*, and representative ERF family proteins**.** Full-length amino acid sequences of OsERF1 (XP_015636763.1), OsERF94 (XP_015635116.1), GhERF1 (AAO59439.1), AtERF1 (NP_188965.1), TaPIE1 (ABU62817.1), SlERF1 (NP_001234848.1), ZmERF1-like (NP_001148691.1), and SbERF039 (XP_002446900.1) were aligned to compare sequence conservation among representative ERF-family members. Shaded residues indicate conserved or highly similar amino acids, with darker shading representing stronger conservation.

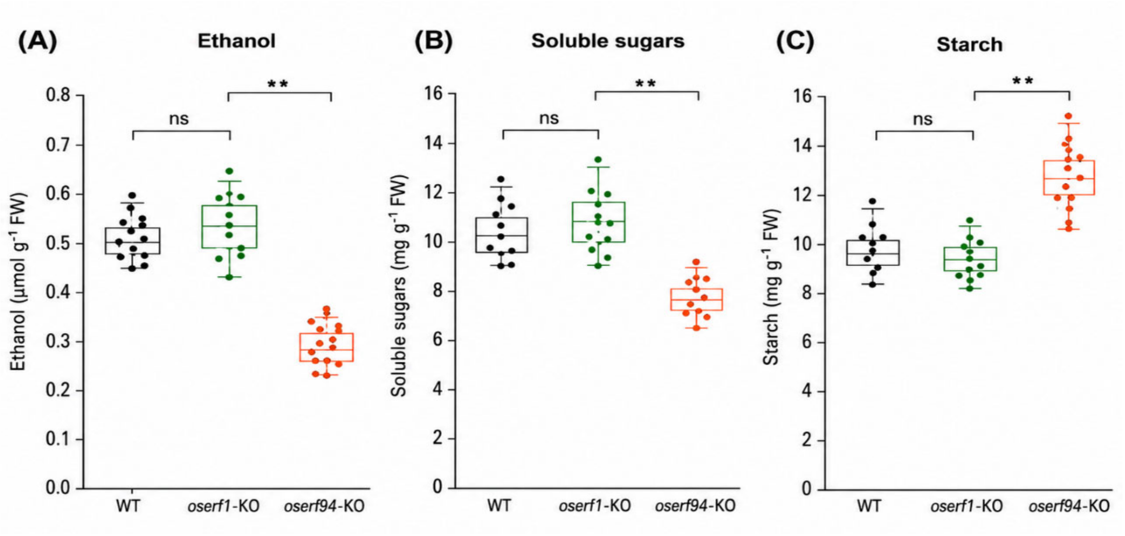

Supplementary Figure S2. Raw data distribution of metabolite contents. Raw data distribution of (**A**) ethanol, (**B**) soluble sugar, and (**C**) starch contents in WT, *oserf1*-KO, and *oserf94*-KO seeds under PHS-inducing conditions. Each dot represents a biological replicate. Boxes show the interquartile range with the median, and whiskers indicate the data range. Statistical significance was assessed relative to WT; ** indicates $p < 0.01$, and ns indicates not significant.

Supplementary Table S1. List of oligonucleotides and primers used in this study.

Primer Name	Sequence (Primer Direction 5′-3′)	Purpose
J67 pBOsC sgSEQ - FW	CAGCTTGGCTCTAGTCGACC	
K20 RGEN scaffold RV	CGGTGCCACTTTTTCAAGTT	
*OsERF1*_sgRNA up	ggcagGGAGCCTGGTGGCCGACATG	
*OsERF1*_sgRNA down	aaacCATGTCGGCCACCAGGCTCCc	Vector construction
*OsERF94*_sgRNA up	ggcagCACCGAGCTCGTTCGGCAGC	
*OsERF94*_sgRNA down	aaacGCTGCCGAACGAGCTCGGTGc	
Nos ter Fw	TTGCGCGCTATATTTTGTTTT	T-DNA confirm
Bar R Rv	CGTCAACCACTACATCGAGA	
*OsERF1*_sgRNA 1st F1	TCGAACACACCACACACTGA	
*OsERF1*_sgRNA 1st R1	GAGGTTGAGGAGGAGGTGGT	
*OsERF1*_sgRNA 2nd F1	acactctttccctacacgacgctcttccgatctgAGCATGTTGCGGAACCAC	
*OsERF1*_sgRNA 2nd R1	gtgactggagttcagacgtgtgctcttccgatctcCTTCACCTCCGCGAACAC	
*OsERF94*_sgRNA 1st F1	CGCAGCATACATGGACTTCC	deep-sequencing
*OsERF94*_sgRNA 1st R1	GCTTACCACTCCGATCACCT	
*OsERF94*_sgRNA 2nd F1	acactctttccctacacgacgctcttccgatctgGATGATCAAGTTCGGTGGCG	
*OsERF94*_sgRNA 2nd R1	gtgactggagttcagacgtgtgctcttccgatctcCTTCGGGTCGCGGATCTC	
O sERF1 Fw	GCTCCTCAACTTCCCGCT	
O sERF1 Rv	TCGCCTCTCTTTCTCCGTTT	
O sERF94 Fw	AGGTGATCGGAGTGGTAAGC	
O sERF94 Rv	CTTCCCAGTACTGCTCCCA	
OsNCED Fw	GGCTACATCCTGTCCTTCGT	
OsNCED Rv	CTCCCTCTGGTCACTTCCTC	
OsABI5 Fw	GTGGTGATGGGGTTCCTGAA	
OsABI5 Rv	ATCTTGGCGTTCTCCTCCTC	qRT-PCR analysis
OsGA3ox2 Fw	ACTCGGGCTTCTTCACCTTC	
OsGA3ox2 Rv	GAGGAAGTAGCCGAGCGAG	
OsADH2 Fw	CGCCATGATATCCTGCTTCG	
OsADH2 Rv	GCTGTGCGTGATGAACTTCT	
OsPDC1 Fw	GAAGCTTCCAGATGACGGC	
OsPDC1 Rv	CCTCAGTCCGGACCTTCTTT	
